# Outcomes and risk factors associated with in-hospital mortality in patients undergoing coronary artery bypass grafting with low ejection fraction

**DOI:** 10.3389/fcvm.2024.1513149

**Published:** 2025-02-13

**Authors:** Yang Zhao, Xu Yu, Xiaolong Ma, Liang Zhang, Zehui Wang

**Affiliations:** ^1^ Department of Cardiovascular Surgery, Anhui Chest Hospital, Hefei, Anhui, China; ^2^ Department of Cardiac Surgery, Beijing Anzhen Hospital, Beijing Institute of Heart Lung and Blood Vessel Diseases, Capital Medical University Beijing, Beijing, China; ^3^ Depertment of Cardiology, The First Hospital of Shanxi Medical University, Taiyuan, Shanxi, China; ^4^ Shanxi Medical University, Taiyuan, Shanxi, China; ^5^ Shanxi Innovation Center for Integrated Management of Hypertension, Hyperlipidemia and Hyperglycemia Correlated with Cardiovascular and Cerebrovascular Diseases, Taiyuan, Shanxi, China

**Keywords:** coronary artery bypass grafting, cardiac insufficiency, in-hospital mortality, risk factors, LVEF

## Abstract

**Objective:**

To investigate the short-term prognosis and risk factors associated with in-hospital mortality in patients undergoing coronary artery bypass grafting (CABG) with low ejection fraction.

**Methods:**

Clinical data were collected from 765 patients who underwent CABG with an ejection fraction of less than 40% between 2019 and 2023 at Anhui Chest Hospital and Beijing Anzhen Hospital, Capital Medical University. The patients were categorized into a in-hospital mortality group (*n* = 38) and a in-hospital survival group (*n* = 727), based on whether they died within 30 days post-operation. Univariate and multivariate logistic regression analyses were employed to identify risk factors for in-hospital mortality. The relationship between these risk factors and the likelihood of in-hospital mortality was assessed using restricted cubic splines (RCS). Additionally, predictive values were evaluated by plotting receiver operating characteristic curves (ROC).

**Results:**

In-hospital mortality occurred in 38 out of the 765 patients, resulting in an incidence rate of 4.97%. Compared to the survival group, those in the mortality group exhibited significantly higher rates of exploratory thoracotomy, intra-aortic balloon pump usage, extracorporeal membrane oxygenation application, gastrointestinal bleeding incidents, and acute renal failure occurrences. Independent risk factors for in-hospital mortality included preoperative age, left ventricular ejection fraction (LVEF), fasting glucose levels (Glu), and glomerular filtration rate (eGFR). Conversely, standardized preoperative administration of oral nitrates and aspirin as well as intraoperative utilization of internal mammary arteries emerged as protective factors against in-hospital mortality. ROC analysis revealed predictive efficiencies for age at 68.5%, LVEF at 76.6%, Glu at 60.5%, while eGFR demonstrated a predictive efficiency of 78.1%.

**Conclusion:**

The incidence of in-hospital mortality in patients undergoing coronary artery bypass grafting with low ejection fraction is correlated with several factors, including advanced age, LVEF, Glu, eGFR, and the standardized preoperative administration of oral nitrates and aspirin. These findings serve as a guide for enhancing the in-hospital prognosis for this patient population in clinical practice.

## Introduction

Low LVEF in the context of coronary artery disease (CAD) is defined as CAD accompanied by an LVEF of less than 40%, with the reduced LVEF attributed to ischemic cardiomyopathy. Although surgical interventions such as CABG have significantly improved the prognosis for these patients (1, 2), in-hospital mortality rates and complication incidences remain alarmingly high, resulting in relatively poor outcomes (3–6).

As the prevalence of CAD continues to rise, so too does the number of patients presenting with low LVEF (7). For this patient population, it is crucial to identify risk factors early on, as they play a significant role in clinical decision-making and ensuring appropriate medical support for critically ill individuals. Therefore, this study aims to investigate the short-term prognosis and risk factors associated with in-hospital mortality in patients undergoing CABG with low LVEF.

## 2 Methods and materials

### 2.1 Study population

The study consecutively enrolled 765 patients undergoing CABG surgery from January 2019 to December 2023 at both Anhui Chest Hospital and Beijing Anzhen Hospital. Based on the occurrence of in-hospital mortality, patients were categorized into two groups: the in-hospital death group (*N* = 38) and the in-hospital survival group (*N* = 727). In-hospital mortality was defined as the incidence of death among patients during their hospitalization.

### 2.2 Inclusion and exclusion criteria

***Inclusion Criteria:***

1. Patients who have undergone CABG surgery,
2. Patients aged over 18 years,
3. Patients with transesophageal echocardiography (TEE) or transthoracic echocardiography (TTE) assessment indicating a LVEF of less than 40%.

***Exclusion Criteria:***

1. Patients diagnosed with hypothyroid heart disease,
2. Patients experiencing acute decompensated heart failure,
3. Patients requiring emergency surgical intervention,
4. Patients with a history of cardiac arrest or cardiopulmonary resuscitation and those exhibiting bradycardia defined as a heart rate of fewer than 50 beats per minute.

### 2.3 Study design and data collection

This study conducted a retrospective collection and analysis of patients' baseline data, encompassing factors such as sex, age, body mass index (BMI), medical history (including hypertension, diabetes, hyperglycemia, stroke, and myocardial infarction), NYHA classification, echocardiographic parameters (LVEF and E/A ratios), prior medications, preoperative laboratory assessments, and procedural details. Additionally, information regarding patients' complications and the utilization of mechanical support devices was also gathered.

### 2.4 Ethic approval

This study was conducted in accordance with the Declaration of Helsink. The studies involving human participants were reviewed and approved by Anhui Chest Hospital and Beijing Anzhen Hospital of ethics committee. The studies were conducted in accordance with the local legislation and institutional requirements. The participants provided their written informed consent to participate in this study.

### 2.5 Statistical analysis

Statistical analyses were performed using R version 4.3. Numerical variables are presented as mean ± standard deviation, while categorical variables are expressed as frequency (percentage). For inter-group comparisons, either the chi-square test or Fisher's exact test was employed. Data exhibiting a skewed distribution are reported as median (inter-quartile range), with the Mann-Whitney *U* test utilized for inter-group comparison. Logistic regression analysis was conducted to identify factors influencing in-hospital mortality, incorporating gender, age, BMI, history of hypertension, history of diabetes, and troponin levels as covariates in subsequent multivariate logistic regression models. ROCwere generated for illustrative purposes. Additionally, RCS were applied to examine the linear relationship between risk factors and in-hospital death. A *P*-value of less than 0.05 was considered statistically significant.

## 3 Results

### 3.1 Patients' characteristics

As presented in Table 1, among the 765 patients studied, there were 553 males (72.29%), with a mean age of 62 years (ranging from 55 to 68 years). A total of 38 cases (4.97%) resulted in in-hospital mortality. In comparison to surviving patients, those who died during hospitalization were older, with increased prevalence of stroke history, elevated plasma glucose levels, preoperative troponin levels, and preoperative CK-MB levels. Additionally, a greater proportion underwent on-pump CABG surgery (all *P* < 0.05). Patients who experienced in-hospital death demonstrated lower LVEF, reduced usage of ACE inhibitors (ACEI), angiotensin receptor blockers (ARB), statins, nitrate ester medications, as well as diminished estimated glomerular filtration rate (eGFR) levels and lower utilization of left internal mammary artery grafts (LIMA) compared to survivors (all *P* < 0.05).

**Table 1:** Patients' detailed characteristics.

	In-hospital survival (*n* = 727)	In-hospital death (*n* = 38)	*P* value
Age (years)	62.00 (55.00–67.00)	67.50 (65.00–69.00)	0.004
Male *n* (%)	532 (73.18%)	21 (55.26%)	0.016
NYHA Grades III-IV *n* (%)	323 (44.43%)	21 (55.26%)	0.191
Hypertension *n* (%)	354 (48.69%)	22 (57.89%)	0.269
Diabetes *n* (%)	331 (45.53%)	22 (57.89%)	0.136
Hyperlipidemia *n* (%)	293 (40.30%)	16 (42.11%)	0.825
History of stroke *n* (%)	113 (15.54%)	11 (28.95%)	0.029
History of myocardial infarction *n* (%)	269 (37.00%)	10 (26.32%)	0.182
Moderate or severe mitral regurgitation *n* (%)	117 (16.09%)	8 (21.05%)	0.42
Moderate or severe aortic regurgitation *n* (%)	11 (1.51%)	1 (2.63%)	0.589
Concomitant ventricular aneurysm *n* (%)	165 (22.70%)	4 (10.53%)	0.078
Oral medications before surgery			
ACE inhibitors and ARBs *n* (%)	378 (51.99%)	13 (34.21%)	0.033
*β* receptor blockers *n* (%)	592 (81.43%)	30 (78.95%)	0.702
Statins *n* (%)	613 (84.32%)	27 (71.05%)	0.031
Nitrates *n* (%)	678 (93.26%)	25 (65.79%)	<0.001
Aspirin *n* (%)	491 (67.54%)	20 (52.63%)	0.057
Preoperative ultrasound			
LVEF (%)	38.00 (35.00–40.00)	32.50 (28.00–35.00)	<0.001
E/A	0.76 (0.60–1.06)	0.75 (0.57–1.44)	0.793
Preoperative tests			
eGFR (ml/min/1.73 m^2^)	82.74 (74.34–96.54)	69.80 (57.80–82.13)	<0.001
Glu (mmol/l)	7.44 (5.61–9.29)	8.15 (5.87–13.24)	0.047
Triglycerides (mmol/l)	1.68 (1.16–1.82)	1.60 (1.15–1.68)	0.6
Cholesterol (mmol//l)	3.95 (3.35–4.35)	3.95 (3.25–4.06)	0.403
Troponin(ng/ml）	0.22 (0.07–1.21)	1.26 (0.17–2.48)	<0.001
CK-MB (U/L)	3.00 (1.80–8.11)	5.50 (2.12–15.75)	0.018
BNP (pg/ml)	344.00 (154.00–639.50)	318.50 (203.50–1,287.75)	0.173
Surgical information			
Use the LIMA *n* (%)	458 (63.00%)	10 (26.32%)	<0.001
Cardiopulmonary bypass *n* (%)	181 (24.89%)	15 (39.47%)	0.045

### 3.2 Primary in-hospital outcome for patients with CABG and low LVEF

As illustrated in Table 2, patients who suffered in-hospital mortality had significantly higher rates of exploratory thoracotomy, intra-aortic balloon pump placement, extracorporeal membrane oxygenation use, gastrointestinal bleeding incidents, and acute renal failure occurrences when compared to surviving patients (all *P* < 0.05).

**Table 2:** Comparison of short-term outcomes between patients in in-hospital death group and patients in in-hospital survival group.

Variables	In-hospital survival (*n* = 727)	In-hospital death (*n* = 38)	*P* value
Exploratory thoracotomy	23 (3.16%)	13 (34.21%)	<0.001
IABP	172 (23.66%)	29 (76.32%)	<0.001
ECMO	10 (1.38%)	10 (26.32%)	<0.001
Gastrointestinal bleeding	11 (1.51%)	3 (7.89%)	0.004
Acute renal failure	21 (2.89%)	13 (34.21%)	<0.001

### 3.3 Impact of cardiopulmonary bypass (CPB) on short-term outcome of patients with CABG and low LVEF

According to Table 3 findings, patients undergoing on-pump CABG exhibited a higher rate of in-hospital mortality than those receiving off-pump CABG procedures (*P* < 0.05). No significant differences were noted regarding exploratory thoracotomy rates or the incidence of intra-aortic balloon pump use; extracorporeal membrane oxygenation application; gastrointestinal bleeding events; or acute renal failure occurrences between the two groups (all *P* > 0.05).

**Table 3:** Comparison of short-term outcome between off-pump and on-pump patients.

	Off pump (*n* = 569)	On pump (*n* = 196)	*P* value
Exploratory thoracotomy	26 (4.57%)	10 (5.10%)	0.761
IABP	147 (25.83%)	51 (26.02%)	0.959
ECMO	15 (2.64%)	5 (2.55%)	0.949
Gastrointestinal bleeding	8 (1.41%)	6 (3.06%)	0.136
Acute renal failure	21 (3.69%)	13 (6.63%)	0.085
In-hospital mortality	23 (4.04%)	15 (7.65%)	0.045

### 3.4 Logistic analysis for risk factors of in-hospital mortality of patients with CABG and low LVEF

As presented in Table 4, several risk or protective factors were screened via uni-variate logistic regression analysis. High age (OR = 1.10, 95% CI: 1.04–1.16, *P* = 0.002), high in-hospital Glu levels (OR = 1.14, 95% CI: 1.06–1.24, *P* < 0.001), high in-hospital CK-MB levels (OR = 1.02, 95% CI: 1.00–1.03, *P* = 0.010), low LVEF (OR = 0.84, 95% CI: 0.79–0.90, *P* < 0.001) and low eGFR (OR = 0.97, 95% CI: 0.95–0.98, *P* < 0.001) were screened as risk factors. Male gender (OR = 0.35, 95% CI: 0.15–0.81, *P* = 0.014), prior administration of nitrates (OR = 0.12, 95% CI: 0.05–0.28, *P* < 0.001), aspirin therapy (OR = 0.41, 95% CI: 0.18–0.91, *P* = 0.029), and the use of LIMA (OR = 0.28, 95% CI: 0.12–0.65, *P* = 0.003) were screened as protective factors.

**Table 4:** Risk factor analysis for patients' in-hospital mortality.

Variables	Uni-variate-logistic			Multiple-variate-logistic		
	OR	95% CI	*P*	OR	95% CI	*P*
Age	1.10	1.04–1.16	0.002	1.10	1.05–1.16	<0.001
Male	0.35	0.15–0.81	0.014	0.53	0.25–1.10	0.088
NYHA Grades III-IV	1.35	0.61–2.99	0.465			
Hypertension	1.87	0.82–4.27	0.140			
Diabetes	1.83	0.82–4.12	0.142			
Hyperlipidemia	1.25	0.57–2.74	0.584			
History of stroke	1.56	0.61–4.00	0.357			
History of myocardial infarction	1.36	0.92–1.66	0.064			
Moderate or severe mitral regurgitation	0.70	0.20–2.38	0.567			
Moderate or severe aortic regurgitation	2.72	0.32–22.97	0.358			
Concomitant ventricular aneurysm	0.21	0.03–1.56	0.126			
ACE inhibitors and ARBs	0.80	0.36–1.78	0.584			
β receptor blockers	0.89	0.35–2.28	0.812			
Statins	0.44	0.19–1.02	0.054			
Nitrates	0.12	0.05–0.28	<0.001	0.15	0.06–0.38	<0.001
Aspirin	0.41	0.18–0.91	0.029	0.43	0.20–0.93	0.032
LVEF	0.84	0.79–0.90	<0.001	0.83	0.77–0.88	<0.001
E/A	1.43	0.77–2.64	0.254			
eGFR	0.97	0.95–0.98	<0.001	0.97	0.95–0.99	<0.001
Glu	1.14	1.06–1.24	<0.001	1.10	1.01–1.21	0.030
Triglycerides	1.14	0.79–1.64	0.497			
Cholesterol	0.86	0.58–1.28	0.460			
Troponin	1.06	0.99–1.13	0.097			
CK-MB	1.02	1.00–1.03	0.010	1.01	1.00–1.02	0.162
BNP	1.00	1.00–1.00	0.065			
Use of LIMA	0.28	0.12–0.65	0.003	0.39	0.17–0.90	0.028

Further multi-variate logstics analysis identified several significant independent risk factors for in-hospital mortality: high age (OR: 1.10, 95% CI: 1.05–1.16, *P* < 0.001), high in-hospital Glu levels (OR: 1.10, 95% CI: 1.01–1.21, *P* = 0.030), low LVEF (OR: 0.83, 95% CI: 0.77–0.88, *P* < 0.001), and low eGFR (OR: 0.97, 95% CI: 0.95–0.99, *P* < 0.001). The use of LIMA (OR: 0.39, 95% CI: 0.17–0.90, *P* = .028), prior administration of nitrate esters [OR: .15; [CI]: .06–.38; [*P*] < .001], and aspirin therapy [OR: .43; [CI]: .20–.93; [*P*] = .032] were found to be significantly protective factors against in-hospital mortality.

### 3.5 RCS analysis for risk factors of in-hospital mortality

**Figure 1:**
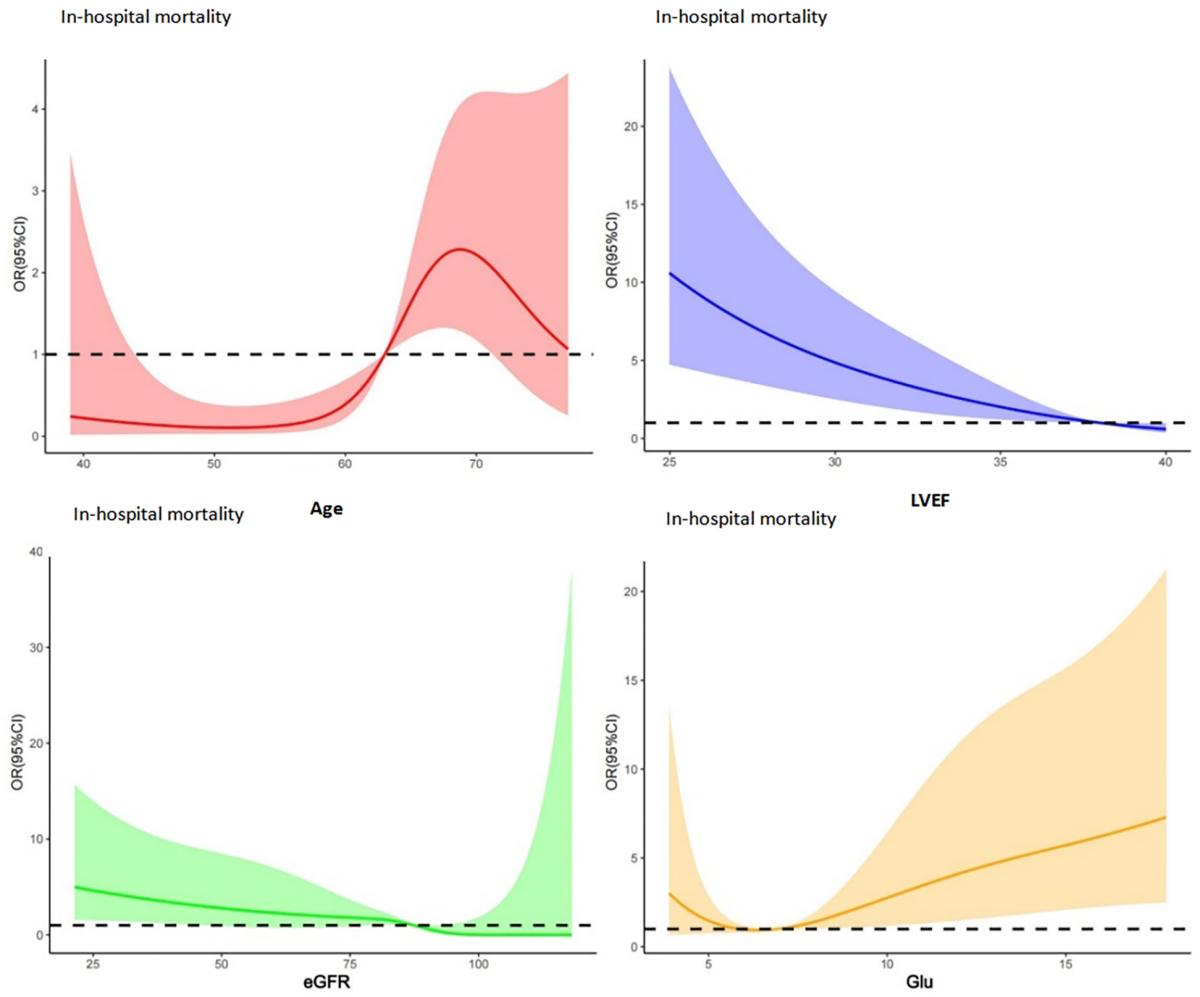
RCS analysis results for patients' in-hospital mortality: red: RCS for age; blue: RCS for LVEF; green: RCS for eGFR; yellow: RCS for Glu, and the identified cut-off values include age >63 years, eGFR <82 ml/min, GLU >6.7 mmol/l, and LVEF <38%.

As illustrated in Figure 1, the RCS analysis indicated that age, LVEF, eGFR, and GLU are non-linearly associated with in-hospital mortality. The identified cut-off values include age >63 years, eGFR <82 ml/min, GLU >6.7 mmol/l, and LVEF <38%.

### 3.6 ROC curve analysis for risk factors of in-hospital morality

**Figure 2:**
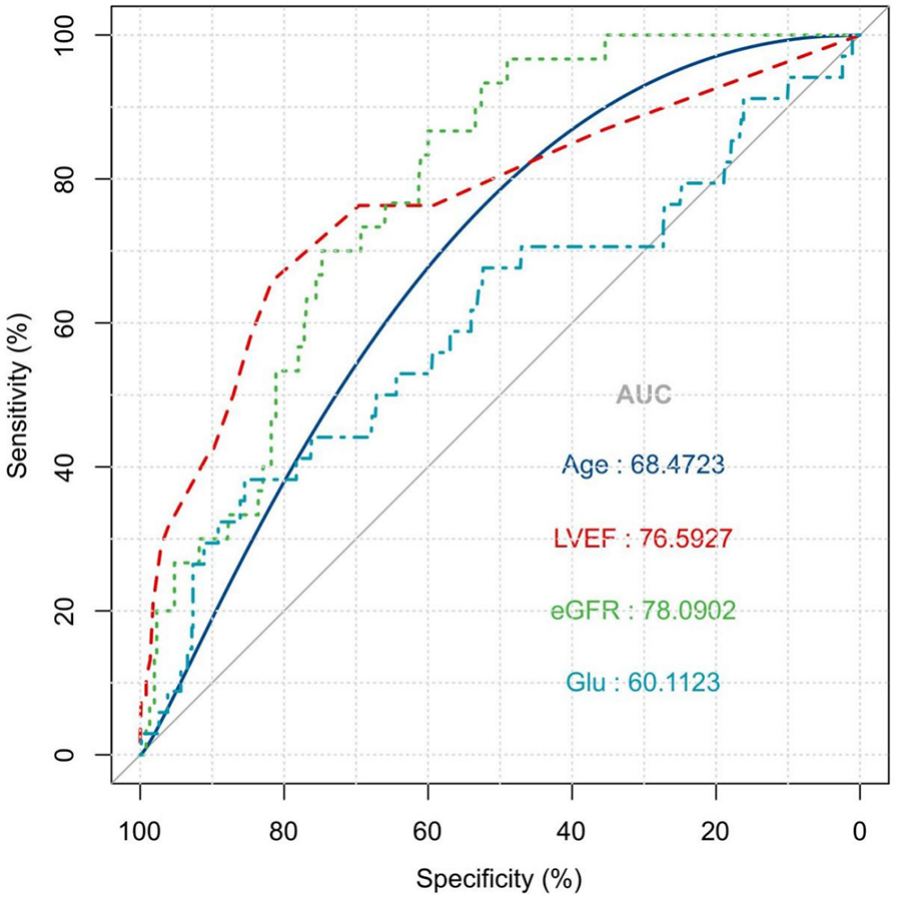
ROC curve for risk factor of patients' in-hospital mortality: the ROC curve analysis indicated that the area under the ROC curve for age, LVEF, eGFR, and GLU were 68.5%, 76.6%, 78.1%, and 60.1%, respectively.

As illustrated in Figure 2, the ROC curve analysis indicated that the area under the ROC curve for age, LVEF, eGFR, and Glu were 68.5%, 76.6%, 78.1%, and 60.1%, respectively, which indicated that a moderate diagnositic value of LVEF and eGFR and relative low diagnositic value of age and Glu. In addition, the Hosmer-Lemeshow goodness-of-fit statistic *P*-value was 0.944, which valided the fit of the model.

## 4 Discussion

This retrospective study analyzes the outcomes and risk factors associated with in-hospital mortality among patients undergoing CABG with low LVEF. The results indicate that patients with low LVEF exhibit a poorer prognosis compared to those with normal LVEF. In our cohort, the in-hospital mortality rate was 4.97%, which aligns closely with other reported cohorts worldwide (8–10), and is significantly higher than that of patients with normal LVEF who underwent CABG surgery (11). This disparity can be attributed to coronary stenosis leading to acute or chronic ischemic myocardial damage and severe remodeling of the left ventricle. Consequently, these patients often present with compromised cardiac functional reserve, categorizing them as a high-risk population for CABG surgery. Given this context, it is crucial to conduct comprehensive preoperative risk stratification for these patients beyond merely focusing on their LVEF levels. Therefore, this study aims to provide valuable insights for clinical practice concerning CABG candidates with low LVEF.

To date, guidelines recommend CABG as the preferred revascularization strategy for patients presenting with low LVEF combined with multivessel disease (1, 2). Numerous large clinical cohorts have demonstrated that compared to those receiving pharmacological therapy and percutaneous coronary intervention (PCI), CABG offers greater benefits (11–13). However, due to an increased prevalence of myocardial hibernation and diminished myocardial activity post-procedure, these patients remain at elevated risk following CABG revascularization. Our findings corroborate this notion through a higher incidence of in-hospital gastrointestinal bleeding (1.8%) and acute kidney injury (4.4%) observed in our study (14–16). Additionally, we noted a notably high utilization rate of intra-aortic balloon pump (IABP) support at 26.2%, which substantially exceeds the average IABP usage rate of approximately 5% seen in other populations (17).

Previous studies have indicated that advanced age is a significant factor influencing recovery in patients with low LVEF post-CABG (18–20). Older patients typically exhibit lower cardiac reserve and often present with more preoperative comorbidities. After controlling for other confounding variables, our analysis revealed that age remains an independent predictor of in-hospital mortality. The utilization of the LIMA has been shown to significantly enhance long-term survival rates among CABG patients (21). Our findings suggest that LIMA usage plays a protective role against in-hospital mortality, potentially due to its ability to self-regulate blood flow and improve ischemic myocardial perfusion.

The advantages of utilizing the left internal mammary artery (LIMA) in coronary artery bypass grafting (CABG) are as follows: 1. High long-term patency rate; 2. Enhancement of myocardial perfusion and cardiac function; 3. Decrease in mortality rates and the incidence of major adverse cardiovascular events (MACE); 4. Improvement in patients' quality of life. These benefits have been substantiated by numerous studies (22, 23), including our own research, which has arrived at a similar conclusion: for CABG patients with low ejection fraction, LIMA serves an independent protective role for these individuals. From the aspect of cardiac function, it helps to increase LVEF. Studies that followed up patients after CABG found that the recovery of cardiac function in patients who used LIMA was better than that in patients who did not use LIAM. This is because the stable blood flow provided by theLIMA can allow hibernating or stunned myocardium to recover, enhance myocardial contractility, and thereby improve the overall pumping function of the heart (24, 25).

Because patients with CAD and low LVEF exhibit characteristics such as intraoperative hemodynamic instability and often present with secondary mitral valve disease, some individuals may require CABG under cardiopulmonary bypass. Our findings indicate that cardiopulmonary bypass is associated with increased in-hospital mortality in patients with low LVEF, while its impact on other outcomes appears to be less pronounced. Several prior studies have demonstrated that off-pump CABG can enhance short-term outcomes for patients with low LVEF (26, 27). Although CABG may offer short-term advantages in off-pump scenarios, we recommend that surgeons customize their surgical approach based on the severity of each patient's condition and preoperative status.

## 5 Conclusions

In conclusion, the in-hospital risk for patients undergoing CABG with low LVEF is significantly elevated compared to routine CABG surgery performed on patients with normal LVEF. This study identified age, LVEF, Glu, and eGFR as independent risk factors predictive of in-hospital mortality. Conversely, regular administration of nitrate esters, aspirin use, and utilization of LIMA were found to be independent protective factors against in-hospital mortality. Further prospective studies involving larger sample sizes are warranted to validate our findings.

## Data Availability

The raw data supporting the conclusions of this article will be made available by the authors, without undue reservation.
